# Crystal structure of a new phen­yl(morpholino)methane­thione derivative: 4-[(morpholin-4-yl)carbothioyl]benzoic acid

**DOI:** 10.1107/S2056989020003977

**Published:** 2020-03-27

**Authors:** Finagnon Hyacinthe Agnimonhan, El-Eulmi Bendeif, Léon Ahoussi Akanni, Ahokannou Fernand Gbaguidi, Eddy Martin, Emmanuel Wenger, Claude Lecomte

**Affiliations:** a Laboratoire de Chimie Organique Physique et de Synthèse., Faculté des Sciences et Techniques (FAST), Université Abomey-Calavi, BP 526 Cotonou, Benin; b CRM2, UMR CNRS 7036, Université de Lorraine, F-54506, Vandoeuvre-lès-Nancy, France; c Bruker France SAS, 4 allée Lorentz Champs sur Marne, 77447 Marne la Vallée Cedex 2, France; d CRM2 UMR CNRS 7036, Université de Lorraine, F-54506, Vandoeuvre-lès-Nancy, France

**Keywords:** crystal structure, phen­yl(morpholino)­methane­thione

## Abstract

4-[(Morpholin-4-yl)carbothioyl]benzoic acid, C_12_H_13_NO_3_S, a novel phen­yl(morpholino)methane­thione derivative, crystallizes in the monoclinic space group *P*2_1_/*n*. The morpholine ring adopts a chair conformation and the carb­oxy­lic acid group is bent out slightly from the benzene ring mean plane. The mol­ecular geometry of the carb­oxy­lic group is characterized by similar C—O bond lengths [1.266 (2) and 1.268 (2) Å] as the carboxyl­ate H atom is disordered over two positions. This mol­ecular arrangement leads to the formation of dimers through strong and centrosymmetric low barrier O—H⋯O hydrogen bonds between the carb­oxy­lic groups. In addition to these inter­molecular inter­actions, the crystal packing consists of two different mol­ecular sheets with an angle between their mean planes of 64.4 (2)°. The cohesion between the different layers is ensured by C—H⋯S and C—H⋯O inter­actions.

## Abstract 2

The first mol­ecular compound synthesized and crystallized in Benin is reported. This work was carried out during the first crystallographic training session at the X-TechLab in Sèmè City, organized within the IUCr–UNESCO OpenLab framework.

## Chemical context   

There is intense research inter­est in developing phen­yl(morpholino)­methane­thione derivatives for pharmaceutical applications. They inhibit the activity of the enzymes MGL (mono­acyl­glycerol lipase) and FAAH (fatty acid amide hydro­lase) (Kapanda *et al.*, 2009 ▸; Draoui, 2009 ▸). MGL and FAAH respectively catalyse the degradation reactions of anandamide and 2-arachidonoylglycerol (2-AG) (Mechoulam *et al.*, 1995 ▸), which are endocannabinoids with beneficial effects in pathophysiological phenomena such as anxiety and pain, and neurodegenerative diseases such as Alzheimer’s (Walker *et al.*, 2000 ▸; Scherma *et al.*, 2008 ▸; Zvonok *et al.*, 2008 ▸). In a continuation of our work on the synthesis of phen­yl(morpholino)­methane­thione derivatives (Agnimonhan *et al.*, 2017 ▸), we report herein the synthesis and crystal structure analysis of a new compound, 4-[(morpholin-4-yl)carbothioyl]benzoic acid.
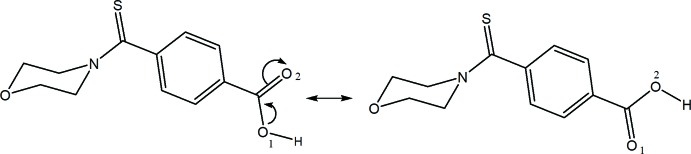


## Structural commentary   

**Figure 1:**
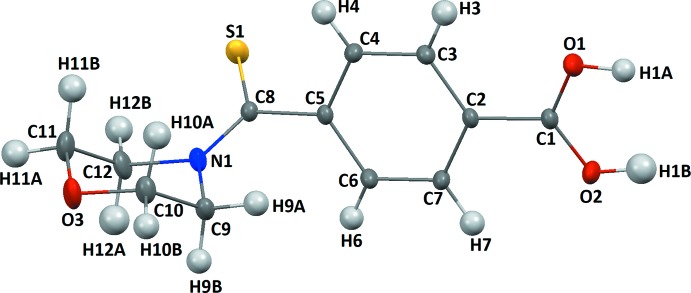
A view of 4-(morpholine-4-carbono­thio­yl) benzoic acid with the atom-numbering scheme. Displacement ellipsoids are drawn at the 50% probability level.

**Figure 2:**
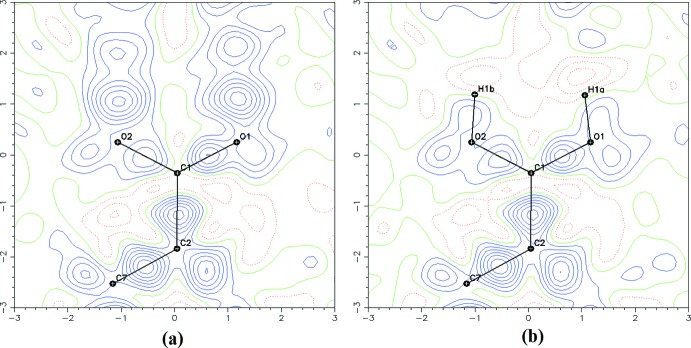
Residual electron density maps in the dimer COO plane calculated at the end of the independent atom refinement: (*a*) without the contribution of the hydrogen atom of the carb­oxy­lic group and (*b*) with the contribution of the hydrogen atom of the carb­oxy­lic group. The contour level is 0.05 e Å^−3^.

The title compound (Fig. 1 ▸) crystallizes in the monoclinic space group *P*2_1_/*n* with four mol­ecules in the unit cell (*Z* = 4). The hydrogen-atom coordinates were located using the high-quality residual electron density maps (Fig. 2 ▸), which also show the bonding electrons and oxygen lone pairs. The mol­ecular structure is not planar, as shown in Fig. 1 ▸. The morpholine ring adopts a chair conformation. The torsion angle between the morpholine group and the phenyl ring around C5—C8 (C thio­amide) is 3.49 (2)°. Such a conformation of the morpholine ring was also observed in the crystal structure of 2-meth­oxy-*N*-(morpholin-4-yl­carb­ono­thio­yl) benzohydrazide hemihydrate (Singh *et al.*, 2007 ▸). The carb­oxy­lic acid group is bent slightly [0.15 (2) Å] out of the plane of the aromatic ring. The electron density deformation map calculated without the contribution of the carb­oxy­lic hydrogen (Fig. 2 ▸ *a*) shows that this carb­oxy­lic H atom is split over two positions H1*A* and H1*B*, linked respectively to atoms O1 and to O2 with a refined population of 0.54 (4)/0.46 (4). This disorder is confirmed by the resulting residual map (Fig. 2 ▸ *b*) and by the equivalent C—O1 [1.266 (2) Å] and C—O2 [1.268 (2) Å] bond lengths. As expected, these distances are significantly longer than classical C=O bonds [1.210 (8) Å] and are shorter than conventional C—O—H [1.311 (2) Å] bonds (Allen, 2002 ▸; Groom *et al.*, 2016 ▸). Given the fact that the obtained results are averaged over the time scale and space of the experiments, the distribution of the electronic density reflects the superposition of the two configurations associated with the disorder (flipping) of the hydrogen atom of the carboxylic group. Thus, the hydrogen atom is shared *via* a double-well hydrogen bond, which leads to equivalent C—O bond lengths

## Supra­molecular features   

**Figure 3:**
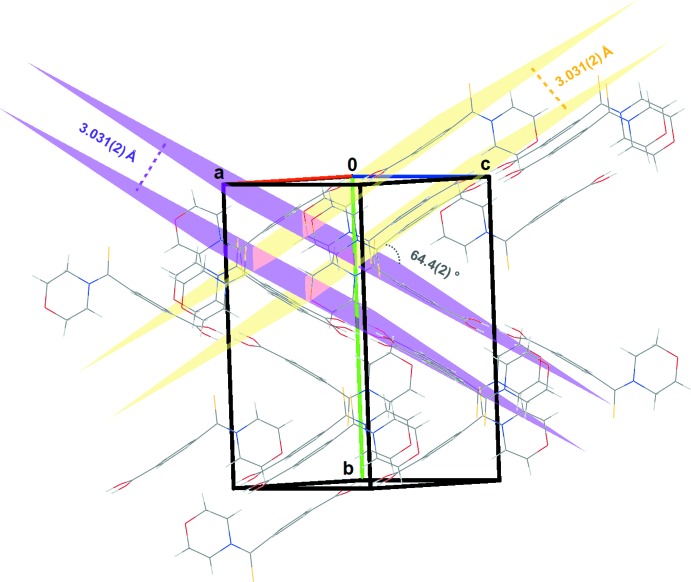
A packing diagram for the title compound viewed along the [101] direction, showing the arrangement of two different mol­ecular sheets.

**Figure 4:**
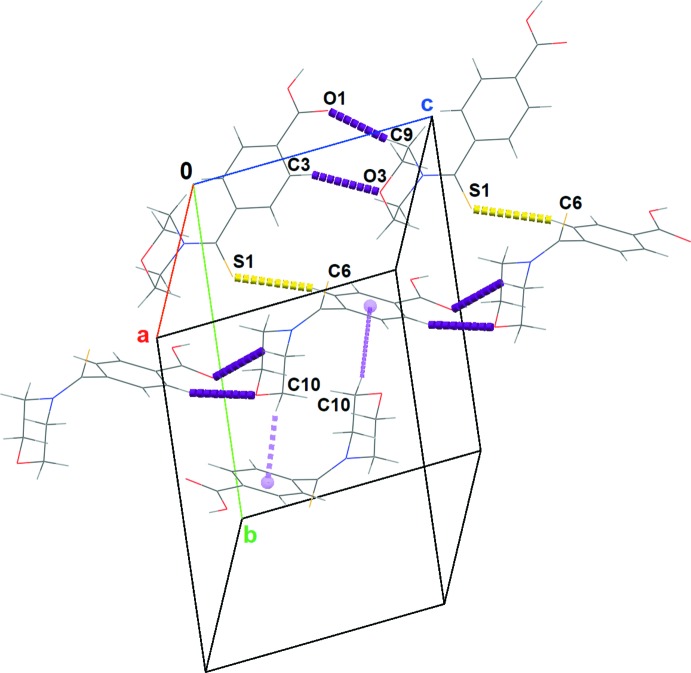
A projection of the three-dimensional network of inter­molecular inter­actions (purple and yellow dotted lines) of the title compound. The C—H⋯π inter­actions are shown with violet dashed lines.

The crystal packing (Fig. 3 ▸) consists of two different mol­ecular sheets. The angle between the mean planes of the two sheets is 64.4 (2)° and the intra-sheet distance is 3.031 (2) Å. The building block is a centrosymmetric dimer built from strong and centrosymmetric double-well low-barrier O—H⋯O hydrogen bonds between two COOH groups. It is worth noting that the carb­oxy­lic groups are inter­connected in a head-to-head fashion with significantly short O1⋯O2 inter­action [2.666 (1) Å]. Gilli & Gilli (2000 ▸) have documented such hydrogen bonds and similar features were also discussed by Benali-Cherif *et al.* (2014 ▸) in their work on polymorphs of *para-*amino benzoic acid. The dimers are themselves connected *via* weak inter­molecular C—H⋯O and C—H⋯S inter­actions (Table 1 ▸, Fig. 4 ▸). Besides these short contacts, C—H⋯π inter­actions occur between the sheets, leading to a highly linked three-dimensional network of inter­molecular inter­actions (Fig. 4 ▸).

## Database survey   

An unsubstituted analogue of the title compound has previously been reported, *viz.* morpholin-4-yl(phen­yl)methane­thione (Guntreddi *et al.*, 2014 ▸; Chen *et al.*, 2016 ▸). Similar structures with a planar nucleus and a chair conformation around the methane­thione group have also been reported, including 1-(4-chloro­thio­benzo­yl)piperidine (Muth­u­raj *et al.*, 2007 ▸), piperidin-1-yl(pyridin-4-yl)methane­thione (Ray *et al.*, 2013 ▸), ferrocen-1-yl(morpholin-4-yl)methane­thione (Patra *et al.*, 2013 ▸) and (3,5-dimethyl-1*H*-pyrazo-1-yl)(morpholin-4-yl)methane­thione (El-Sayed *et al.*, 2018 ▸).

## Synthesis and crystallization   

All reagents along with the used solvent were obtained from Sigma–Adrich, Prolabo and Acros Organic and used without further purification. To a mixture of 4-formyl­benzoic acid (0.75 g; 5 mmol) and morpholine (0.63 ml, 7.5 mmol) in di­methyl­formamide (15 ml) under agitation was added montmorillonite K-10 (0.35 g) and sulfur S_8_ (0.26 g, 8 mmol). The brown mixture obtained was irradiated in a microwave for 10–15 minutes at 940 W. The temperature of the reaction mixture was in the range 411–416 K. After cooling to room temperature, the mixture was poured into a solution of ethyl acetate and hydro­chloric acid (0.1 *M*, 100 ml) to eliminate the excess of sulfur and amine. It was then saturated with an NH_4_Cl solution and finally washed with distilled water (2 × 100 ml); the organic phase obtained was dried over MgSO_4_ before being concentrated by evaporation. Brown prismatic crystals suitable for single-crystal X-ray analysis were grown by slow evaporation from an ethanol solution at ambient temperature in the presence of air or in the freezer. The synthesized crystals were stable in air and highly soluble in polar organic solvent (*e.g*. ethyl acetate, dimethyl sulfoxide).

## Refinement   

Crystal data, data collection and structure refinement details are summarized in Table 2 ▸. All hydrogen atoms were clearly identified in difference-Fourier maps and their atomic coordinates and isotropic displacement parameters were refined. At the end of refinement, the hydrogen atom of the carb­oxy­lic group was localized in the Fourier maps and refined accordingly by splitting its position on two sites with a refined occupancy ratio of 0.54 (4)/0.46 (4).

The quality of this room-temperature (298 K) crystal structure is also indicated by the experimental electron density deformation maps calculated after the IAM refinement at 0.75 Å^−1^ experimental resolution: they are of excellent quality (see Fig. 2 ▸). They show detailed features in the electron density distribution in the chemical bonds (0.35 e Å^−3^ for a C—C bond), electron density lone pairs and almost no noise. This surprising data quality is mostly due to the quality of the detector and to the high redundancy of the experiment [22827 collected I(H), 3664 unique reflections, most of them (3317) having [*I* > 2σ(*I*)].

## Supplementary Material

Crystal structure: contains datablock(s) global, I. DOI: 10.1107/S2056989020003977/dx2024sup1.cif

Structure factors: contains datablock(s) I. DOI: 10.1107/S2056989020003977/dx2024Isup2.hkl

Supporting information file. DOI: 10.1107/S2056989020003977/dx2024Isup3.cml

CCDC reference: 1991487

Additional supporting information:  crystallographic information; 3D view; checkCIF report

## Figures and Tables

**Table 1:** Hydrogen-bond geometry (Å, °) *Cg* is the centroid of the C2–C7 ring.

*D*—H⋯*A*	*D*—H	H⋯*A*	*D*⋯*A*	*D*—H⋯*A*
C9—H9*B*⋯O1^i^	0.98 (2)	2.47 (2)	3.2985 (19)	142.1 (16)
C12—H12*B*⋯S1	0.99 (3)	2.55 (2)	3.0860 (18)	113.8 (17)
O2—H1*B*⋯O1^ii^	0.93 (2)	1.75 (2)	2.6661 (15)	165 (5)
O1—H1*A*⋯O2^ii^	0.93 (2)	1.78 (2)	2.6661 (15)	160 (4)
C3—H3⋯O3^iii^	0.94 (2)	2.64 (2)	3.536 (2)	158 (2)
C6—H6⋯S1^iv^	0.89 (2)	2.996 (2)	3.8650 (14)	166 (2)
C10—H10*B*⋯*Cg* ^v^	1.00 (2)	2.74 (2)	3.6180 (18)	147 (2)

Symmetry codes: (i) 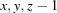; (ii) 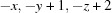; (iii) 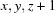; (iv) 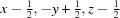; (v) 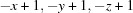.

**Table 2:** Experimental details

Crystal data	
Chemical formula	C_12_H_13_NO_3_S
*M*_r_	251.29
Crystal system, space group	Monoclinic, *P*2_1_/*n*
Temperature (K)	293
*a*, *b*, *c* (Å)	8.3252 (1), 17.1485 (3), 9.3505 (1)
β (°)	116.249 (1)
*V* (Å^3^)	1197.26 (3)
*Z*	4
Radiation type	Mo *K*α
μ (mm^−1^)	0.27
Crystal size (mm)	0.15 × 0.15 × 0.08

Data collection	
Diffractometer	Bruker D8 Quest
Absorption correction	Multi-scan (*SADABS*; Bruker, 2019 ▸)
*T*_min_, *T*_max_	0.959, 0.981
No. of measured, independent and observed [*I* > 2σ(*I*)] reflections	22827, 3664, 3317
*R*_int_	0.024
(sin θ/λ)_max_ (Å^−1^)	0.715

Refinement	
*R*[*F* ^2^ > 2σ(*F* ^2^)], *wR*(*F* ^2^), *S*	0.048, 0.131, 1.08
No. of reflections	3664
No. of parameters	211
No. of restraints	2
H-atom treatment	All H-atom parameters refined
Δρ_max_, Δρ_min_ (e Å^−3^)	0.55, −0.48

Computer programs: *APEX3* and *SAINT* (Bruker, 2019 ▸), SHELXT2014 (Sheldrick, 2015*a* ▸), *SHELXL2014* (Sheldrick, 2015*b* ▸), *PLATON* (Spek, 2020 ▸), *Mercury* (Macrae *et al.*, 2020 ▸), *enCIFer* (Allen *et al.*, 2004 ▸) and *WinGX* (Farrugia, 2012 ▸).

## supplementary crystallographic information

### Crystal data

**Table:**

C_12_H_13_NO_3_S	*F*(000) = 528
*M**_r_* = 251.29	*D*_x_ = 1.394 Mg m^−^^3^
Monoclinic, *P*2_1_/*n*	Mo *K*α radiation, λ = 0.71073 Å
*a* = 8.3252 (1) Å	Cell parameters from 22827 reflections
*b* = 17.1485 (3) Å	θ = 2.4–30.5°
*c* = 9.3505 (1) Å	µ = 0.27 mm^−^^1^
β = 116.249 (1)°	*T* = 293 K
*V* = 1197.26 (3) Å^3^	Prism, brown
*Z* = 4	0.15 × 0.15 × 0.08 mm

### Data collection

**Table:**

Bruker D8 Quest diffractometer	*R*_int_ = 0.024
Radiation source: micro-focus sealed X-ray tube	θ_max_ = 30.5°, θ_min_ = 2.4°
ω scans	*h* = −11→11
Absorption correction: multi-scan (SADABS; Bruker, 2019)	*k* = −24→24
*T*_min_ = 0.959, *T*_max_ = 0.981	*l* = −13→13
22827 measured reflections	60 standard reflections every 120 min
3664 independent reflections	intensity decay: none
3317 reflections with *I* > 2σ(*I*)	

### Refinement

**Table:**

Refinement on *F*^2^	2 restraints
Least-squares matrix: full	Hydrogen site location: difference Fourier map
*R*[*F*^2^ > 2σ(*F*^2^)] = 0.048	All H-atom parameters refined
*wR*(*F*^2^) = 0.131	*w* = 1/[σ^2^(*F*_o_^2^) + (0.0661*P*)^2^ + 0.4174*P*] where *P* = (*F*_o_^2^ + 2*F*_c_^2^)/3
*S* = 1.08	(Δ/σ)_max_ < 0.001
3664 reflections	Δρ_max_ = 0.55 e Å^−^^3^
211 parameters	Δρ_min_ = −0.47 e Å^−^^3^

### Special details

**Table:**

Geometry. All esds (except the esd in the dihedral angle between two l.s. planes) are estimated using the full covariance matrix. The cell esds are taken into account individually in the estimation of esds in distances, angles and torsion angles; correlations between esds in cell parameters are only used when they are defined by crystal symmetry. An approximate (isotropic) treatment of cell esds is used for estimating esds involving l.s. planes.

### Fractional atomic coordinates and isotropic or equivalent isotropic displacement parameters (Å^2^)

**Table:**

	*x*	*y*	*z*	*U*_iso_*/*U*_eq_	Occ. (<1)
N1	0.44570 (18)	0.31569 (7)	0.41547 (15)	0.0357 (3)	
C9	0.4178 (2)	0.39766 (8)	0.36720 (19)	0.0375 (3)	
C10	0.5884 (2)	0.43113 (10)	0.3757 (2)	0.0453 (4)	
C11	0.6791 (3)	0.30807 (11)	0.3284 (3)	0.0539 (4)	
C12	0.5096 (3)	0.27137 (10)	0.3165 (2)	0.0470 (4)	
O3	0.65390 (19)	0.38815 (8)	0.28269 (17)	0.0541 (3)	
H7	−0.016 (3)	0.4221 (12)	0.575 (2)	0.050 (5)*	
H6	0.112 (3)	0.3489 (12)	0.444 (2)	0.046 (5)*	
H4	0.573 (3)	0.3397 (12)	0.835 (2)	0.047 (5)*	
H9A	0.378 (3)	0.4270 (12)	0.438 (2)	0.051 (5)*	
H3	0.452 (3)	0.4107 (13)	0.975 (3)	0.052 (5)*	
H9B	0.324 (3)	0.4011 (12)	0.258 (3)	0.050 (5)*	
H12B	0.532 (3)	0.2169 (15)	0.355 (3)	0.061 (6)*	
H10A	0.679 (3)	0.4269 (14)	0.494 (3)	0.062 (6)*	
H12A	0.412 (4)	0.2743 (15)	0.199 (3)	0.074 (7)*	
H11A	0.716 (3)	0.2808 (14)	0.256 (3)	0.062 (6)*	
H11B	0.783 (3)	0.3030 (14)	0.449 (3)	0.062 (6)*	
H10B	0.569 (3)	0.4855 (13)	0.333 (3)	0.053 (6)*	
H1B	−0.099 (6)	0.496 (3)	0.869 (5)	0.074 (19)*	0.54 (6)
H1A	0.147 (5)	0.509 (2)	1.062 (5)	0.045 (16)*	0.46 (6)
S1	0.47641 (7)	0.19242 (2)	0.59878 (5)	0.04786 (14)	
C2	0.20398 (17)	0.42178 (8)	0.78784 (14)	0.0292 (2)	
C5	0.35519 (17)	0.33605 (7)	0.62500 (15)	0.0283 (2)	
O1	0.21386 (16)	0.48436 (8)	1.01739 (13)	0.0468 (3)	
C1	0.11742 (19)	0.46256 (8)	0.87542 (16)	0.0333 (3)	
O2	−0.05100 (16)	0.47194 (9)	0.80599 (14)	0.0517 (3)	
C4	0.45678 (18)	0.35472 (8)	0.78541 (16)	0.0330 (3)	
C6	0.17845 (18)	0.36164 (9)	0.54576 (16)	0.0343 (3)	
C8	0.43045 (17)	0.28503 (8)	0.53941 (15)	0.0296 (2)	
C3	0.38209 (18)	0.39791 (8)	0.86649 (15)	0.0331 (3)	
C7	0.10388 (18)	0.40414 (9)	0.62725 (16)	0.0343 (3)	

### Atomic displacement parameters (Å^2^)

**Table:**

	*U*^11^	*U*^22^	*U*^33^	*U*^12^	*U*^13^	*U*^23^
N1	0.0492 (7)	0.0297 (5)	0.0396 (6)	0.0039 (5)	0.0299 (5)	−0.0014 (4)
C9	0.0458 (7)	0.0344 (7)	0.0408 (7)	0.0079 (5)	0.0269 (6)	0.0062 (5)
C10	0.0557 (9)	0.0376 (7)	0.0566 (10)	−0.0006 (6)	0.0375 (8)	−0.0012 (7)
C11	0.0636 (11)	0.0508 (9)	0.0685 (12)	0.0107 (8)	0.0486 (10)	−0.0014 (8)
C12	0.0703 (11)	0.0390 (8)	0.0488 (9)	0.0014 (7)	0.0420 (9)	−0.0097 (7)
O3	0.0700 (8)	0.0506 (7)	0.0682 (8)	0.0016 (6)	0.0547 (7)	0.0014 (6)
S1	0.0679 (3)	0.0341 (2)	0.0500 (2)	0.01608 (17)	0.0337 (2)	0.01035 (15)
C2	0.0329 (6)	0.0322 (6)	0.0277 (5)	0.0021 (4)	0.0182 (5)	−0.0003 (4)
C5	0.0311 (6)	0.0297 (6)	0.0292 (5)	0.0014 (4)	0.0181 (5)	0.0000 (4)
O1	0.0478 (6)	0.0604 (7)	0.0327 (5)	0.0117 (5)	0.0184 (5)	−0.0096 (5)
C1	0.0378 (6)	0.0367 (6)	0.0308 (6)	0.0059 (5)	0.0201 (5)	0.0003 (5)
O2	0.0395 (6)	0.0794 (9)	0.0390 (6)	0.0177 (6)	0.0200 (5)	−0.0067 (6)
C4	0.0291 (6)	0.0400 (7)	0.0301 (6)	0.0050 (5)	0.0132 (5)	−0.0006 (5)
C6	0.0309 (6)	0.0474 (7)	0.0255 (5)	0.0028 (5)	0.0134 (5)	−0.0043 (5)
C8	0.0306 (6)	0.0302 (6)	0.0312 (6)	0.0023 (4)	0.0167 (5)	−0.0016 (5)
C3	0.0332 (6)	0.0400 (7)	0.0257 (5)	0.0024 (5)	0.0128 (5)	−0.0028 (5)
C7	0.0281 (6)	0.0466 (7)	0.0299 (6)	0.0056 (5)	0.0145 (5)	−0.0019 (5)

### Geometric parameters (Å, º)

**Table:**

N1—C8	1.3288 (17)	C2—C7	1.3906 (18)
N1—C9	1.4631 (18)	C2—C3	1.3938 (18)
N1—C12	1.4674 (17)	C2—C1	1.4836 (17)
C9—C10	1.501 (2)	C5—C6	1.3937 (18)
C9—H9A	1.00 (2)	C5—C4	1.3948 (18)
C9—H9B	0.98 (2)	C5—C8	1.4973 (17)
C10—O3	1.4202 (19)	O1—C1	1.2664 (17)
C10—H10A	1.03 (2)	O1—H1A	0.926 (19)
C10—H10B	1.00 (2)	C1—O2	1.2681 (18)
C11—O3	1.426 (2)	O2—H1B	0.93 (2)
C11—C12	1.503 (3)	C4—C3	1.3881 (18)
C11—H11A	0.98 (2)	C4—H4	0.91 (2)
C11—H11B	1.08 (2)	C6—C7	1.3841 (18)
C12—H12B	0.99 (3)	C6—H6	0.89 (2)
C12—H12A	1.04 (3)	C3—H3	0.94 (2)
S1—C8	1.6700 (13)	C7—H7	0.95 (2)
			
C8—N1—C9	125.87 (11)	C10—O3—C11	111.19 (13)
C8—N1—C12	123.12 (12)	C7—C2—C3	119.70 (11)
C9—N1—C12	110.80 (12)	C7—C2—C1	119.55 (11)
N1—C9—C10	109.51 (12)	C3—C2—C1	120.68 (11)
N1—C9—H9A	109.5 (12)	C6—C5—C4	119.66 (11)
C10—C9—H9A	110.8 (12)	C6—C5—C8	119.52 (11)
N1—C9—H9B	109.0 (13)	C4—C5—C8	120.72 (11)
C10—C9—H9B	109.6 (12)	C1—O1—H1A	112 (3)
H9A—C9—H9B	108.4 (17)	O1—C1—O2	122.96 (12)
O3—C10—C9	112.31 (14)	O1—C1—C2	118.70 (12)
O3—C10—H10A	109.0 (13)	O2—C1—C2	118.31 (12)
C9—C10—H10A	104.7 (13)	C1—O2—H1B	115 (3)
O3—C10—H10B	105.9 (13)	C3—C4—C5	120.32 (12)
C9—C10—H10B	111.0 (13)	C3—C4—H4	120.4 (13)
H10A—C10—H10B	114.1 (18)	C5—C4—H4	119.3 (13)
O3—C11—C12	111.86 (15)	C7—C6—C5	119.89 (12)
O3—C11—H11A	107.6 (14)	C7—C6—H6	120.1 (13)
C12—C11—H11A	108.8 (14)	C5—C6—H6	119.9 (13)
O3—C11—H11B	109.5 (13)	N1—C8—C5	117.32 (11)
C12—C11—H11B	109.6 (13)	N1—C8—S1	124.75 (10)
H11A—C11—H11B	109.5 (18)	C5—C8—S1	117.81 (9)
N1—C12—C11	109.05 (14)	C4—C3—C2	119.84 (12)
N1—C12—H12B	108.6 (14)	C4—C3—H3	119.8 (13)
C11—C12—H12B	110.5 (13)	C2—C3—H3	120.4 (13)
N1—C12—H12A	108.2 (15)	C6—C7—C2	120.56 (12)
C11—C12—H12A	109.0 (15)	C6—C7—H7	121.0 (13)
H12B—C12—H12A	111.5 (19)	C2—C7—H7	118.5 (13)
			
C8—N1—C9—C10	118.33 (16)	C8—C5—C6—C7	175.39 (13)
C12—N1—C9—C10	−56.57 (18)	C9—N1—C8—C5	8.5 (2)
N1—C9—C10—O3	55.99 (19)	C12—N1—C8—C5	−177.18 (14)
C8—N1—C12—C11	−117.94 (17)	C9—N1—C8—S1	−175.68 (12)
C9—N1—C12—C11	57.12 (19)	C12—N1—C8—S1	−1.4 (2)
O3—C11—C12—N1	−57.0 (2)	C6—C5—C8—N1	64.84 (17)
C9—C10—O3—C11	−56.2 (2)	C4—C5—C8—N1	−118.65 (15)
C12—C11—O3—C10	56.8 (2)	C6—C5—C8—S1	−111.26 (13)
C7—C2—C1—O1	−173.78 (14)	C4—C5—C8—S1	65.25 (15)
C3—C2—C1—O1	9.1 (2)	C5—C4—C3—C2	0.9 (2)
C7—C2—C1—O2	8.3 (2)	C7—C2—C3—C4	−1.9 (2)
C3—C2—C1—O2	−168.82 (14)	C1—C2—C3—C4	175.16 (13)
C6—C5—C4—C3	0.6 (2)	C5—C6—C7—C2	0.1 (2)
C8—C5—C4—C3	−175.88 (13)	C3—C2—C7—C6	1.4 (2)
C4—C5—C6—C7	−1.2 (2)	C1—C2—C7—C6	−175.72 (14)

### Hydrogen-bond geometry (Å, º)

*Cg* is the centroid of the C2–C7 ring.

**Table:**

*D*—H···*A*	*D*—H	H···*A*	*D*···*A*	*D*—H···*A*
C9—H9*B*···O1^i^	0.98 (2)	2.47 (2)	3.2985 (19)	142.1 (16)
C12—H12*B*···S1	0.99 (3)	2.55 (2)	3.0860 (18)	113.8 (17)
O2—H1*B*···O1^ii^	0.93 (2)	1.75 (2)	2.6661 (15)	165 (5)
O1—H1*A*···O2^ii^	0.93 (2)	1.78 (2)	2.6661 (15)	160 (4)
C3—H3···O3^iii^	0.94 (2)	2.64 (2)	3.536 (2)	158 (2)
C6—H6···S1^iv^	0.89 (2)	2.996 (2)	3.8650 (14)	166 (2)
C10—H10*B*···*Cg*^v^	1.00 (2)	2.74 (2)	3.6180 (18)	147 (2)

Symmetry codes: (i) *x*, *y*, *z*−1; (ii) −*x*, −*y*+1, −*z*+2; (iii) *x*, *y*, *z*+1; (iv) *x*−1/2, −*y*+1/2, *z*−1/2; (v) −*x*+1, −*y*+1, −*z*+1.
